# The importance of left ventricular function for long-term outcome after primary percutaneous coronary intervention

**DOI:** 10.1186/1471-2261-8-4

**Published:** 2008-02-23

**Authors:** Pieter A van der Vleuten, Saman Rasoul, Willem Huurnink, Iwan CC van der Horst, Riemer HJA Slart, Stoffer Reiffers, Rudi A Dierckx, René A Tio, Jan Paul Ottervanger, Menko-Jan De Boer, Felix Zijlstra

**Affiliations:** 1 Thoraxcentre, Department of Cardiology, University Medical Centre Groningen, The Netherlands; 2 Department of Cardiology, Isala klinieken, Zwolle, The Netherlands; 3 Department of Nuclear Medicine, Isala klinieken, Zwolle, The Netherlands; 4 Department of Nuclear Medicine and molecular imaging, University Medical Centre Groningen, The Netherlands

## Abstract

**Background:**

In the present study we sought to determine the long-term prognostic value of left ventricular ejection fraction (LVEF), assessed by planar radionuclide ventriculography (PRV), after ST-elevation myocardial infarction (STEMI) treated with primary percutaneous coronary intervention (PPCI).

**Methods:**

In total 925 patients underwent PRV for LVEF assessment after PPCI for myocardial infarction before discharge from the hospital. PRV was performed with a standard dose of 500 Mbq of ^99m^Tc-pertechnetate. Average follow-up time was 2.5 years.

**Results:**

Mean (± SD) age was 60 ± 12 years. Mean (± SD) LVEF was 45.7 ± 12.2 %. 1 year survival was 97.3 % and 3 year survival was 94.2 %. Killip class, multi vessel-disease, previous cardiovascular events, peak creatin kinase and its MB fraction, age and LVEF proved to be univariate predictors of mortality. When entered in a forward conditional Cox regression model age and LVEF were independent predictors of 1 and 3 year mortality.

**Conclusion:**

LVEF assessed by PRV is a powerful independent predictor of long term mortality after PPCI for STEMI.

## Background

The management of patients with an acute ST-elevation myocardial infarction (STEMI) has fundamentally changed over the last twenty years. In the eighties thrombolytic agents were introduced and more recently primary percutaneous coronary intervention (PPCI) has been shown to be even more effective [1,2]. In patients surviving the first days after PPCI, risk stratification is of great clinical relevance for the further (medical) management. Among others, global left ventricular function has always been viewed as an important prognostic factor after acute myocardial infarction. Earlier trials in large cohorts of STEMI-patients, treated with either thrombolytic agents or supportive care (no reperfusion-therapy), have confirmed this prognostic value for a period of six months after myocardial infarction [3-7].

Planar radionuclide ventriculography (PRV) is a well established and widely used technique for the assessment of left ventricular function. The technique is simple, robust and easy to perform [8-10]. PRV assesses LVEF by measurement of photon-activity of the bloodpool in the left ventricle in both the end-diastolic and end-systolic phase of the cardiac cycle. The aim of the present study was to evaluate the long term prognostic value of LVEF, assessed by routine PRV, in a large cohort of patients treated with PPCI for STEMI.

## Methods

As part of two consecutive multicentre randomized controlled trials consecutive patients treated with PPCI for STEMI in two large hospitals in the Netherlands were entered in a registry [11,12]. The registry was opened in April 1998 and was closed in December 2004. The inclusion criteria differed in inclusion of all Killip classes in GIPS 1 [11] versus only Killip 1 in GIPS 2 [12]. Baseline characteristics such as medical history, cardiovascular risk factors, heart rate and blood pressure, delay-times and procedural parameters were recorded. For the present study data from the registries of two large hospitals in The Netherlands were used. Average follow-up time was 2.5 years. No patients were lost to follow-up. The present study was conducted in accordance with the declaration of Helsinki and was approved by the institutional review boards of both cooperating hospitals.

PRV was performed in routine clinical practice before discharge from the hospital, between day 1 and day 11 after myocardial infarction. Four patients with atrial fibrillation were excluded. Measurements were performed using the multiple-gated equilibrium method with in vivo labelling of red blood cells with 99mTc pertechnetate, after pre-treatment with 1 mg. of stannous chloride. A γ-camera (General Electric, Milwaukee, WI, U.S.A.) was used. The camera head was positioned in the best septal LAO projection, typically with a caudal tilt of 5–10 degrees. R-wave triggering was performed in a 20% beat acceptance window with 2/3 forward and 1/3 backward framing per cardiac cycle, for 20 frames per R-R interval for a total of 6 minutes. LVEF was calculated using a Star View computer (General Electric, Wisconsin, USA) using the fully automatic PAGE program (version 2.3). The standard deviation of the difference between repeat measurements obtained by this technique is 1–2% [13].

### Statistical analyses

Analyses were performed with the commercially available package SPSS version 12.0.1 (SPSS inc, Chicago, IL, USA). Continuous data of LVEF values were expressed as mean ± standard deviation (SD). Mortality rates were calculated according to the product-limit method. Further estimation of risk was performed using Cox proportional hazards models. Variables considered as potential predictors for multivariable modelling were selected by univariate analyses and were subsequently selected by stepwise forward selection, with entry and retention in the model set at a significance level of .05.

## Results

PRV was not performed in 14 patients because they were too hemodynamically unstable. Furthermore 10 patients died before PRV could be performed. In total 925 patients underwent routine PRV. Clinical and angiographic characteristics are shown in Table 1. All patients underwent PPCI of the infarct related artery, which was successful in 87.2% (defined as TIMI 3 flow in combination with a myocardial blush grade ≥ 2). PRV was performed at a median of 2 days after PPCI (range 1 day – 11 days). Mean LVEF was 45.7 ± 12.2 % (interquartile-range: 37.0 % – 54.0 %).

**Table 1:** Baseline clinical and angiographic characteristics

Age, yrs (mean ± SD)	59.8 ± 12.0
Male sex	77.8
	
Body mass index, kg/m2 (mean ± SD)	26.7 ± 3.8
	
History of MI	9.9
History of PCI	5.1
History of CABG	2.8
History of stroke	2.8
	
Diabetes mellitus	9.7
Hypertension	28.5
Hyperlipidemia	22.1
Current smoker	50.7
Positive family history	42.3
	
Ischemia duration, min (mean ± SD)*	205 ± 212
	
Killip class 1	95.9
Killip class 2	2.4
Killip class 3	1.3
Killip class 4	0.4
	
Anterior MI	48.6
Multivessel disease	51.4
TIMI 3 flow after PCI	96.9
	
Successful reperfusion‡	87.2
Intra-aortic balloon pump	5.0
Mechanical ventilation	0.5
Stent	57.6
Glycoprotein IIb/IIIa receptor blocker	21.2
	
Max CK, U/l (mean ± SD)	2450 ± 2159
Max CK-MB, U/l (mean ± SD)	248 ± 198

Data are displayed as percentage, unless otherwise indicated. *Ischemia duration denotes time between onset of symptoms and until PCI; ‡successful reperfusion denotes TIMI 3 flow and myocardial blush grade 2 or 3;

CABG = coronary artery bypass grafting

CK = creatin kinase

CK-MB = creatin kinase myoglobin binding

MI = myocardial infarction

PCI = percutaneous coronary intervention

SD = standard deviation

TIMI = thrombolysis in myocardial infarction

**Figure 1:**
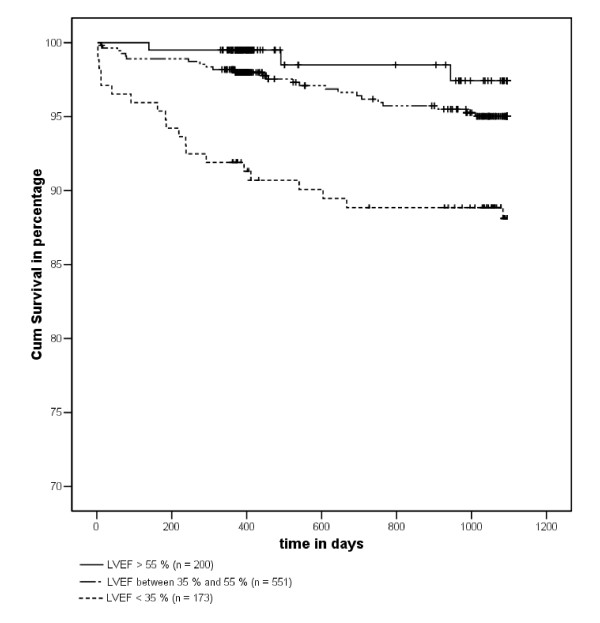
Kaplan-Meier curve of 925 patients who underwent planar radionuclide ventriculography after primary percutaneous coronary intervention for ST-elevation myocardial infarction. LVEF = Left Ventricular Ejection Fraction.

**Figure 2:**
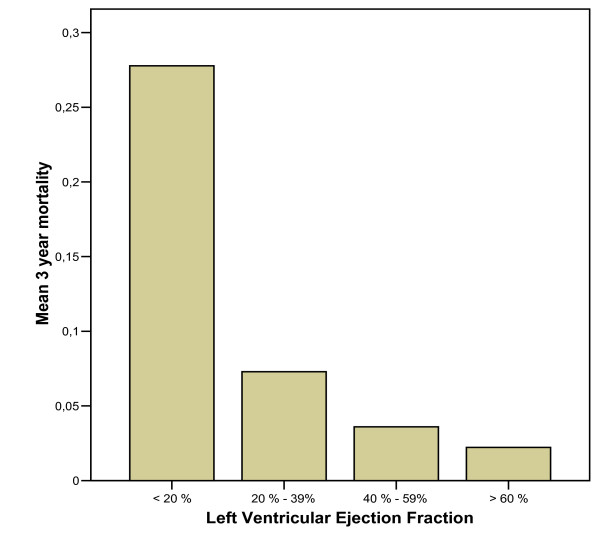
Adjusted 3 year mortality rate for patients who underwent planar radionuclide ventriculography after primary percutaneous coronary intervention for ST-elevation myocardial infarction, grouped by left ventricular ejection fraction.

Follow-up was obtained for all 925 patients. All-cause mortality was 0.2 %, 0.9 %, 2.7 % and 5.8 % at 3 days, 30 days, 1 year and 3 years respectively. Three day mortality in the entire registry was 2.3 %. Kaplan Meier curves for all-cause mortality in the 925 patients who underwent PRV before discharge are shown in Figure 1. The unadjusted mortality rate increased exponentially with decreasing LVEF (Figure 2).

By univariate Cox proportional hazards analysis several baseline clinical characteristics and infarct related parameters were shown to be significant predictors of death. Significant predictors of both 1 year and 3 year mortality were age, history of MI, history of PCI, peak CK, peak CK-MB-fraction and LVEF. Killip class, multivessel disease and history of CABG were only significant univariate predictors of 3 year mortality. Details are shown in Table 2. Sex, history of stroke, diabetes, hypertension, hyperlipidemia, smoking habit, positive family history, infarct-duration, infarct location, TIMI flow after PPCI, myocardial blush grade, use of G2b3a inhibitors, use of intra-aortic balloon pump or mechanical ventilation were not significant predictors of mortality. When a forward conditional Cox proportional hazard model of only the factors age and LVEF was implemented, none of the other variables provided incremental prognostic value (Table 3).

**Table 2:** Predictors of 1 and 3 year mortality by univariate Cox proportional hazard analysis.

*1 year mortality*		
		
Characteristics	Hazard ratio (95% CI)	*p*
Age, per 10 years increase	2.00 (1.35 – 2.97)	0.001
Previous MI	2.91 (1.16 – 7.28)	0.023
Previous PCI	9.58 (4.13 – 22.21)	< 0.001
Max CK, per 500 U/l increase	1.01 (1.00 – 1.02)	0.050
Max CK-MB, per 50 U/l increase	1.09 (1.01 – 1.18)	0.039
LVEF, per 5 % decrease	1.47 (1.25 – 1.73)	< 0.001
		
*3 year mortality*		
		
Characteristics	Hazard ratio (95% CI)	*p*
Age, per 10 years increase	1.63 (1.25 – 2.14)	< 0.001
Previous MI	2.19 (1.06 – 4.52)	0.035
Previous PCI	5.16 (2.50 – 10.7)	< 0.001
Previous CABG	3.27 (1.17 – 9.10)	0.024
Multi-vessel disease	1.50 (1.06 – 2.11)	0.021
Killip class, per class increase	1.73 (1.08 – 2.75)	0.022
Max CK, per 500 U/l increase	1.01 (1.00 – 1.02)	0.040
Max CK-MB, per 50 U/l increase	1.07 (1.01 – 1.14)	0.020
LVEF, per 5 % decrease	1.29 (1.15 – 1.46)	< 0.001

CABG = coronary artery bypass grafting

CK = creatin kinase

CK-MB = creatin kinase myoglobin binding

LVEF = left ventricular ejection fraction

MI = myocardial infarction

PCI = percutaneous coronary intervention

**Table 3:** Predictors of 1 and 3 years mortality by forward conditional Cox proportional hazard analysis.

*1 year mortality*				
				
Characteristics	Hazard ratio	95% CI	Wald χ^2^	*p*
Age, per 10 years	2.01	1.33 – 3.03	11.1	0.001
LVEF, per 5 % decreasing	1.44	1.23 – 1.69	20.4	< 0.001
				
*3 year mortality*				
				
Characteristics	Hazard ratio	95% CI	Wald χ^2^	*p*
Age, per 10 years	1.64	1.25 – 2.15	12.6	< 0.001
LVEF, per 5 % decreasing	1.28	1.14 – 1.44	17.6	< 0.001

LVEF = left ventricular ejection fraction

## Discussion

The present study shows that LVEF assessed shortly after PPCI for STEMI, is a powerful predictor of long term survival. Earlier studies, most designed to establish the value of various pharmacologic interventions after myocardial infarction, have shown the prognostic value of global left ventricular function, measured as LVEF, in terms of mortality and re-admission rates for heart failure [14-17]. However, the follow-up duration and patient selection differed from the present study.

The event-rate was relatively low for a post-infarction cohort, with a 3 year mortality of only 5.8 %. The fact that this study looks at data from patients who underwent PRV on average 2 days after PPCI in the routine of daily clinical practice, in most cases just before discharge or transfer to another hospital, has systematically excluded patients who were too hemodynamically unstable to undergo PRV. For all analyses total mortality was used. It can be hypothesized that the relationship between LVEF and cause-specific mortality would be even stronger. The fact that the traditional risk-factors for coronary artery disease (sex, hypertension, diabetes, hyperlipidemia, smoking and family history) were not significant predictors of mortality may be explained by the fact that these risk-factors for the most part contributed to the occurrence of the index-MI itself and have only limited effect on the prognosis after the index-MI. In addition, a number of these risk-factors (hypertension, hyperlipidemia and smoking) is usually treated more aggressively after the index-MI. The fact that some infarct-treatment parameters, such as use of mechanical ventilation and use of IABP, were not significant predictors of mortality is most likely explained by the relatively low numbers in this cohort with a relatively low event-rate.

Noteworthy is the relatively small difference in prognosis between the patient category with LVEF between 35 % and 55 % and the patient category with LVEF above 55 %, which is generally viewed as the lower limit of normal. In contrast, there was a large difference in survival between the patient category with LVEF between 35 % and 55 % and the patient category with LVEF below 35, which is the current cut-off point for implantable cardioverter defibrillator implementation (Figure 1).

The data in the present study suggest that markers of infarct size, such as maximum creatin kinase myoglobin binding level, Killip class and previous myocardial damage from earlier events add up to a risk burden which is related to global left ventricular function. LVEF can therefore be viewed as a representative of the final common pathway of left ventricular damage when predicting long-term prognosis after PPCI. The fact that this LVEF-assessment can be performed just a few days after the index myocardial infarction facilitates simple and fast risk stratification after PPCI.

Besides PRV, LVEF can be measured by a number of techniques, which all have their own specific advantages and limitations. For instance echocardiography can be performed easily and at low cost. However, the diagnostic accuracy is limited [18]. Nuclear techniques such as positron emission tomography and single photon emission computed tomography have better diagnostic accuracy, but are more labour intensive and are not available in every hospital. Recently, multi detector row computed tomography has been propagated as very fast and accurate technique for LVEF assessment [19]. However, besides ionising radiation, this technique also requires the use of intravenous nephrotoxic contrast agents. LVEF can even be assessed directly after PPCI by contrast ventriculography. Besides the obvious advantage of almost instant LVEF-assessment, the main drawbacks from this approach are the relatively high volume of nephrotoxic contrast, the limited accuracy and the fact that LVEF can be severely underestimated by myocardial stunning shortly after STEMI. Magnetic resonance imaging is regarded by many to be the gold standard for LVEF measurement [20]. Unfortunately, this technique is limited to patients without intra-corporal devices such as pacemakers and is not generally available for routine clinical patients.

## Conclusion

In conclusion, LVEF assessed by PRV before discharge from the hospital is a powerful independent predictor of long term prognosis after PPCI for STEMI.

## Abbreviations

CABG = Coronary artery bypass grafting, CK = Creatin kinase, CK-MB = Creatin kinase myocardial band, LVEF = Left ventricular ejection fraction, PCI = Percutaneous coronary intervention, PPCI = Primary percutaneous coronary intervention, PRV = Planar radionuclide ventriculography, SD = Standard deviation, STEMI = ST-elevation myocardial infarction, TIMI = Thrombolysis in myocardial infarction (study group).

## Competing interests

The author(s) declare that they have no competing interests.

## Authors' contributions

PVV contributed in data-collection, data-analysis and drafting the manuscript.

SR contributed in data-collection, data-analysis and drafting the manuscript.

WH contributed in PRV acquisition in Zwolle and drafting the manuscript.

ICH contributed in designing the study, data-analysis and drafting the manuscript.

RHS contributed in PRV acquisition in Groningen and drafting the manuscript.

SR contributed in designing the study, data-analysis and drafting the manuscript.

RAD contributed in data-analysis and drafting the manuscript.

RAT contributed in study-design, data-analysis and drafting the manuscript.

JPO contributed in study-design and drafting the manuscript.

MJB contributed in study-design and drafting the manuscript.

FZ contributed in study-design, data-analysis and drafting the manuscript.

All authors have read and approved the final version of the manuscript.

## Pre-publication history

The pre-publication history for this paper can be accessed here:


